# Potential of dual orexin receptor antagonists to prevent Alzheimer’s disease

**DOI:** 10.1002/alz.090830

**Published:** 2025-01-03

**Authors:** Brendan P Lucey, Erik S. Musiek

**Affiliations:** ^1^ Hope Center for Neurological Disorders, St Louis, MO USA; ^2^ Washington University School of Medicine, St Louis, MO USA; ^3^ Washington University School of Medicine, Saint Louis, MO USA; ^4^ Knight Alzheimer Disease Research Center, Saint Louis, MO USA

## Abstract

Sleep disturbances, such as insomnia, are associated with Alzheimer’s disease pathology and future risk of cognitive impairment. This raises the exciting possibility of repurposing existing drugs to prevent or delay Alzheimer’s disease since there are multiple drug approved by the Food and Drug Administration for the treatment of insomnia. Dual orexin receptor antagonists (DORAs) are one such class of medications. The orexin system regulates sleep‐wake activity and individuals with orexin deficiency have narcolepsy, a sleep disorder resulting in excessive daytime sleepiness, sleep paralysis, sleep‐related hallucinations, and cataplexy. Based on its role in narcolepsy pathophysiology, DORAs were developed as a treatment of insomnia. Growing evidence suggests that DORAs may be effective at preventing Alzheimer’s disease pathology. In amyloid precursor protein (APP) transgenic mice that develop amyloid deposition, treatment with a DORA decreased the soluble amyloid‐beta concentrations and prevented amyloid deposition. Recent evidence in humans shows that a DORA, suvorexant, acutely decreases amyloid‐beta and phosphorylated tau levels in human cerebrospinal fluid within hours. In this presentation, we will review recent advances testing the effects of DORAs on biomarkers for Alzheimer’s disease pathology, examine additional potential mechanisms that DORAs may reduce Alzheimer’s disease pathology, and discuss current knowledge gaps regarding the effects of other sleep drugs on Alzheimer’s disease biomarkers. Finally, we will discuss on‐going studies planned to test the effects of DORAs in preclinical animal models and humans to lay the groundwork for future phase III clinical trials to prevent or delay the onset of symptomatic Alzheimer’s disease.

